# Acute Macular Neuroretinopathy after Intravitreal Ozurdex Injection in a Patient with Birdshot Chorioretinopathy: A Case Report

**DOI:** 10.1159/cop/acaag025

**Published:** 2026-09-16

**Authors:** Niku Dhillon, David Knight

**Affiliations:** Department of Ophthalmology, National Treatment Centre Highland, Inverness, UK

**Keywords:** Acute macular neuroretinopathy, Birdshot chorioretinopathy, Ozurdex, Case report

## Abstract

**Introduction:**

Acute macular neuroretinopathy (AMN) is a rare retinal disorder linked to ischaemia of the deep capillary plexus. It has been reported after intravitreal injections, and this report describes an unreported temporal association between intravitreal Ozurdex and AMN.

**Case presentation:**

An HLA-A29-positive female with Birdshot chorioretinopathy and recurrent cystoid macular oedema, treated with prednisolone, mycophenolate mofetil, and adalimumab, presented with a paracentral ring scotoma within 24 h of a third Ozurdex injection. Multimodal imaging and 10-2 perimetry were performed and monitored over 6 months. Examination showed 6/15 vision and parafoveal pallor. OCT demonstrated foveal-sparing outer retinal hyper-reflectivity corresponding to the scotoma. Multimodal imaging outlined the area of retinal injury. Visual fields confirmed a ring scotoma. At 6 months, symptoms persisted and OCT showed photoreceptor loss.

**Conclusion:**

This case describes a previously unreported temporal association between intravitreal Ozurdex and AMN. Ophthalmologists should be aware of rare sight threatening events following intravitreal therapy including corticosteroids.

## Introduction

Acute macular neuroretinopathy (AMN) is a rare macular disorder characterised by an acute onset scotoma, often close to fixation. The underlying mechanism of retinal injury is attributed to microangiopathic ischaemia at the level of the deep retinal capillary plexus, which perfuses the outer retina. AMN was first described as wedge shaped parafoveal lesions pointing towards the fovea [1], corresponding to watershed zones supplied by terminal arterioles, which are susceptible to vascular compromise.

Multimodal imaging can help the diagnosis AMN, particularly when the fundus appears unremarkable. Hallmark OCT findings are localised to the outer retina manifesting as hyper-reflective bands within the outer plexiform and outer nuclear layers, which often resolve leaving photoreceptor disruption [2]. Risk factors are vaso-occlusive in nature and include viral illness, oral contraception, vasoconstrictive agents, caffeine, epinephrine, pregnancy, thrombocytopaenia, and anaemia [3, 4].

Visual recovery is variable and complete recovery uncommon [5]. In a review by Turberville et al., approximately half of patients failed to regain vision, whereas 29% showed some improvement. Currently, no recognised treatment exists for AMN.

AMN has been documented following intravitreal delivery of anti-VEGF [6, 7] medication and triamcinolone acetonide [8]. To our knowledge, we report the first case of AMN occurring after intravitreal Ozurdex injection.

## Case Report

A 55-year-old HLA-A29-positive female undergoing treatment for active Birdshot chorioretinopathy (BSCR) and recurrent cystoid macular oedema (CMO) with prednisolone 5 mg, mycophenolate mofetil 1 g twice daily and fortnightly adalimumab 40 mg injections, presented to the eye casualty with a paracentral ring scotoma, which occurred within 6 h of receiving a third intravitreal Ozurdex.

The patient did not have any recognised risk factors for AMN including no cardiovascular risk factors, normotensive, non-smoker, no recent infections or vaccinations, vasoactive medications or hormone replacement therapy. She was non-vitrectomised and there was no other significant previous ocular history.

Her visual acuity had improved from 6/18 to 6/15. Intraocular pressure (IOP) increased from 12 mm Hg, immediately prior to the Ozurdex injection, to 18 mm Hg at presentation to the eye casualty. No IOP was checked immediately following the injection. Her anterior segment and vitreous showed no inflammation.

Fundus examination revealed a subtle parafoveal ring of pallor and macular OCT demonstrated foveal-sparing band-like hyper-reflectivity within the outer retinal layers, correlating to the patient’s scotoma. Multimodal imaging is described in Figure 1.

**Fig. 1.**
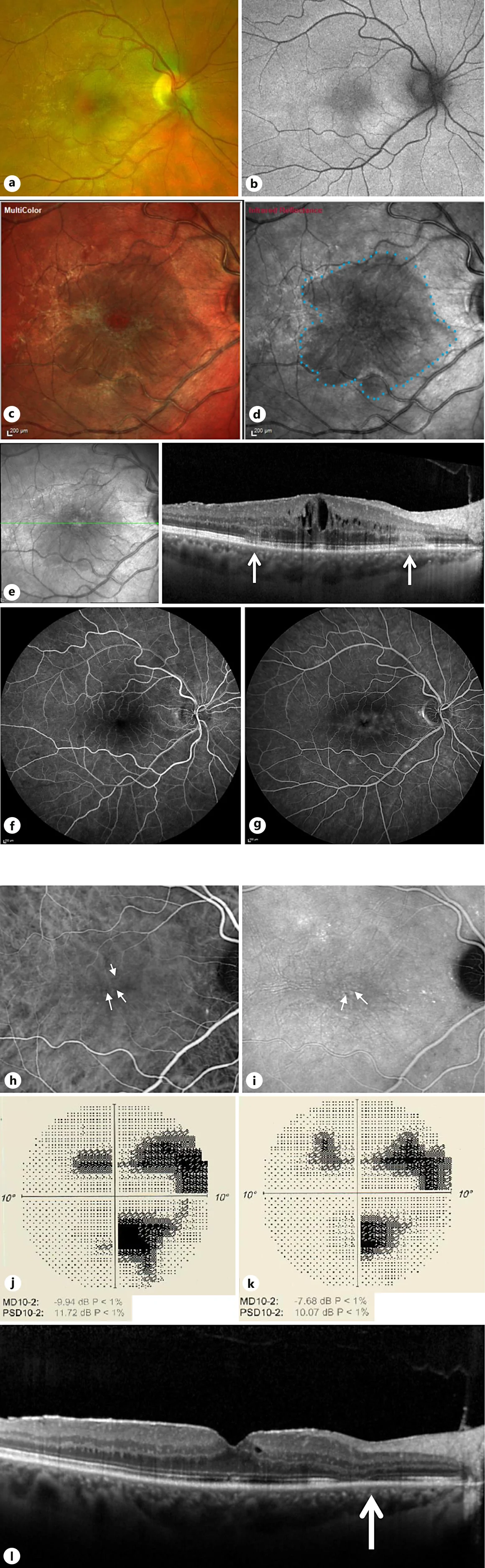
**a** Colour photo shows a ring of subtle parafoveal pallor. **b** Autofluorescence showed a corresponding hyperautofluorescenct ring. Multicolour (**c**) and infrared images (**d**) outline the affected macular area. **e** Macular OCT shows foveal-sparing band-like hyper-reflectivity involving the outer retinal layers, corresponding to the patient’s scotoma. Early-phase FFA (**f**) and late-phase FFA (**g**) showed CMO and capillary leakage. ICGA demonstrated small hypofluorescent foveal spots in mid (**h**) and late-phase (**i**) images. **j** 10-2 visual field confirmed a ring scotoma at presentation. **k** 10-2 visual field showed the density of the scotoma reduced over time but did not fully resolve at 6 months. **l** Macular OCT at 6 months demonstrated resolution of the CMO and hyper-reflective bands, but with photoreceptor loss at the site of injury. OCT, optical coherence tomography; CMO, cystoid macular oedema; FFA, fundus fluorescein angiography; ICGA, indocyanine green angiography.

At 6 months follow-up, the patient remained symptomatic with vision of 6/12. Macular OCT(l) showed resolution of both CMO and hyper-reflective bands; however, photoreceptor loss was observed at the site of injury.

## Discussion

To our knowledge, this is the first reported case of AMN secondary to an intravitreal Ozurdex injection. The close temporal association, with symptom onset within 6 h of the injection, suggests a possible relationship.

Maleki et al. [8] describe a similar case of an HLA-A29-positive BSCR patient receiving mycophenolate and adalimumab who developed AMN following intravitreal triamcinolone acetonide for CMO. They observed visual improvement with G. brimonidine three times daily, which the authors attributed to both brimonidine’s IOP lowering effect and potential enhancement of retinal perfusion. The authors did not establish a mechanism for the corticosteroid induced response.

The exact mechanism of AMN in this case is uncertain and likely multifactorial. Possible mechanisms have been considered in the literature for AMN following an intravitreal procedure. However, from this patient’s clinical presentation, no convincing mechanism can be established.

1. A sudden IOP rise post injection following a well-tunnelled scleral wound without reflux may have reduced ocular perfusion pressure and compromised macular arteriole perfusion. Barash et al. [9] demonstrated reduced macular perfusion, on OCTA, following acute IOP elevation immediately after intravitreal anti-VEGF injections. However, in this case, no post injection pressure was taken to establish a clinically meaningful pressure-mediated mechanism.
2. Conversely, acute hypotony related to a poorly tunnelled scleral wound may have resulted in a transient retinal or choroidal circulatory disturbance. Masaoka et al. [10] reported hypofluorescent areas on indocyanine green angiography (ICGA) in eyes with traumatic hypotony maculopathy, supporting the concept that acute hypotony can alter posterior segment perfusion. No clinical signs of hypotony were recorded in this case.
3. The underlying inflammatory nature of BSCR may predispose to a transient vasculitic occlusion at the level of the deep capillary plexus. While AMN has not been directly attributed to BSCR, Carey et al. [11] reported a case of BSCR which presented as paracentral acute middle maculopathy, a related retinal disorder, which affects the inner nuclear layer. From the available data, we cannot demonstrate an inflammatory vascular event.
4. Mechanical trauma related to implant delivery should also be considered. Implants propelled at high velocity to strike the retina causing injury including vitreous haemorrhage have been reported [12]. However, in this case, no direct implant-retina contact nor vitreous haemorrhage was observed.

Sinha et al. [13] describe how multimodal imaging can aid diagnosis, with near infrared reflectance and OCT being most sensitive. OCTA was not performed in this case, although we recognise the value of this tool in identifying reduced perfusion in the deep capillary plexus and could have strengthened the diagnosis.

Fundus autofluorescence showed a ring of hyperautofluorescence (b) corresponding to the macular injury, suggesting RPE stress secondary to an outer retinal insult. Typical ICGA findings in AMN include early- and mid-phase hypofluoresence corresponding to ischaemic areas [14]. Other studies have reported no abnormalities on ICGA including a case series of AMN presentations attributed to COVID-19 infection [15]. We observed late hypofluorescent foveal spots, which would be in keeping with previous ICGA findings, suggesting both the choriocapillaris and outer retina can be implicated in AMN. These findings, however, are not specific enough to establish the mechanism of injury in this case.

This case report was prepared in accordance with the CARE reporting guidelines and the completed CARE Checklist is provided as online supplementary material (for all online suppl. material, see https://doi.org/10.1159/cop/acaag025).

## Conclusion

To our knowledge, this is the first reported case of AMN following intravitreal Ozurdex injection. Although the close proximity in time suggests the injection acted as a precipitating event, in the absence of an established mechanism and the rarity of such a presentation despite widespread use of Ozurdex, means the association should be interpreted cautiously. Multimodal imaging can help the clinician diagnose AMN particularly in an apparently normal looking fundus. This case can be used as a flag for further observation rather than evidence of a confirmed Ozurdex induced complication.

## Statement of Ethics

This study was conducted in accordance with the principles of the Declaration of Helsinki. Ethical approval is not required for this study in accordance with local guidelines as this manuscript is a case report. Written informed consent was obtained from the patient for publication of the details of their medical case and any accompanying images.

## Conflict of Interest Statement

The authors declare no potential conflicts of interest with respect to the research, authorship, and/or publication of this article.

## Funding Sources

No funding was received for this study.

## Author Contributions

N.D. and D.K. contributed to the conception, drafting, and revision of the case report. Both have approved the final version for submission.

## Data Availability

The data that support the findings of this study are not publicly available due to privacy reasons but are available from the corresponding author upon reasonable request.
